# Three-dimensional assessment of obturation volume in lateral canals after three obturation techniques with bioceramic sealer: an in vitro comparative study

**DOI:** 10.1038/s41405-024-00240-5

**Published:** 2024-06-17

**Authors:** Wahid Juha, Elizabeth Sarkis, Yasser Alsayed Tolibah

**Affiliations:** 1 Department of Endodontics, Aleppo University, Aleppo, Syria; 2 Department of Pediatric Dentistry, Damascus University, Damascus, P.O. Box 3062 Syria

**Keywords:** Oral diseases, Anatomy

## Abstract

**Objective:**

This study aimed to evaluate the obturation ability of simulated lateral canal in mandibular premolars at three levels (3, 5, and 7 mm) from the apex using gutta-percha and BC Sealer HiFlow (BCHiF) with different obturation techniques, including continuous wave compaction (CWC), cold lateral condensation (CLC), and single cone (SC) techniques, by a 3D assessment method of the obturation volume with cone beam computed tomography (CBCT) and MIMICS software analysis.

**Methods:**

Thirty intact human mandibular premolars were decoronated, instrumented up to #30 taper 4%, and uniformly irrigated with 5.25% NaOCl and 17% EDTA. Six simulated lateral canals (3 pairs) were prepared at 3, 5, and 7 mm from the apex in each root, using #10 modified C-file. CBCT images were obtained, and lateral canal volumes were calculated using MIMICS software. The samples were divided into three groups: CWC (*n* = 10), CLC (*n* = 10), and SC (*n* = 10). All groups were obturated with BCHiF and gutta-percha. Another CBCT image was taken post-obturation, and 3D lateral canal obturation volume percentages were calculated using MIMICS software. Data were analyzed using SPSS software with One-way ANOVA and Sidak tests (α = 0.05).

**Results:**

Significant differences were observed in the 3D lateral canal obturation volume percentage at all three levels (*P* < 0.05). Both CWC and CLC techniques demonstrated higher 3D lateral canal obturation volume percentages ($\bar{x}$ = 89.64% and $\bar{x}$  =  73.28%; respectively) compared to the SC group) $\bar{x}$  =  43.10%).

**Conclusion:**

BCHiF combined with the CWC technique has a higher ability to achieve preferable 3D obturation volume in the simulated lateral canal at 3, 5, and 7 mm.

**Clinical relevance:**

In cases requiring endodontic treatment with lateral canals, the CWC obturation technique using BCHiF with gutta-percha may offer better outcomes compared to other obturation techniques.

## Introduction

Root canal treatment aims to thoroughly disinfect and seal the root canal system, including the main canals and associated irregularities and lateral canals, which may serve as communication channels between the main canal and the external root surface [1].

The prevalence of lateral canals ranges from 27.4% to 99%, with lower premolars exhibiting the highest proportion [2]. Studies have reported that mandibular premolars can have lateral canals ranging from 40% to 65% [3, 4]. The presence of lateral canals is strongly associated with infection within root canals and the development of apical periodontitis, which can complicate endodontic procedures and affect treatment outcomes [1–5]. Proper chemical cleaning with irrigants and activation systems and then the use of suitable endodontic sealers are crucial for achieving complete sealing of the root canal system [6].

Sealer flow and fluidity are essential characteristics for adequate penetration into the root canal system and dentinal tubules, ensuring effective sealing [7]. Endosequence BC Sealer (Brasseler, Savannah, GA, USA) is a calcium silicate-based sealer known for its stable chemical bond with hydroxyapatite in root dentin and compatibility with the single cone (SC) obturation technique [8]. However, its use with thermal obturation techniques is debatable. A newer formulation, Endosequence BC Sealer HiFlow (BCHiF), was introduced in 2018 to address these concerns, offering lower viscosity when heated and improved radiopacity [9].

BCHiF demonstrates favorable properties such as high calcium ion release, alkaline pH, low cytotoxicity, and good flow characteristics, particularly under high temperatures [10, 11]. Studies have shown that BCHiF with continuous wave compaction (CWC) obturation technique achieves excellent dentinal tubule penetration and effective root canal obturation [12].

While cold lateral condensation (CLC) and CWC are traditional obturation techniques, they may be time-consuming and require specialized equipment [13]. The SC technique has emerged as a simpler alternative, especially when using calcium silicate sealers, and is less operator-dependent [14]. However, it may result in voids in irregularly shaped canals [15].

Cone-beam computed tomography (CBCT) is a valuable imaging tool in endodontics, offering high-resolution three-dimensional visualization without significant radiation exposure [16]. It allows for precise assessment of root canal anatomy and treatment outcomes [17]. Although CBCT has been demonstrated to give an accurate detection, significant and clearer results as structures can be viewed in a 3-dimensional (3D) format [16].

The Materialize Interactive Medical Image Control System (MIMICS) is an advanced image processing software developed by Materialize NV, a Belgian company renowned for its expertise in additive manufacturing software and technology across medical, dental, and other industries. MIMICS is employed for creating 3D designs, generating 3D surface models, performing 3D measurements, and analyzing images derived from stacks of 2D image data, such as CT and CBCT scans [16]. Moreover, the use of CBCT images with the MIMICS software has become common in studies focusing on laboratory investigations related to endodontics [18].

To the best of the researcher’s knowledge, there was a lack of studies that focused on the ability of BCHiF to obturate the lateral canals. Therefore, this study aimed to evaluate the obturation ability of simulated lateral canal in mandibular premolars at three levels (3, 5, and 7 mm) from the apex using gutta-percha BCHiF with continuous wave compaction (CWC), cold lateral condensation (CLC), or single cone (SC) techniques, by a 3D assessment method of the obturation volume with cone beam computed tomography (CBCT) and 3D MIMICS analysis.

The null hypothesis posits no significant differences in 3D lateral canal obturation volume percentage among CLC, CWC, and SC techniques when using BCHF and gutta-percha.

## Materials and methods

### Ethical statement

This experimental in-vitro study was conducted respecting the ethical guidelines of the Declaration of Helsinki. The research project was ethically approved by the Local Research Ethics Committee (UDDS-26-23022021/SRC-132), and was self-funded. Patients had provided informed consent that their extracted teeth would undergo in the current study.

### Sample size calculation

Drawing from the findings of a previous study [2], the sample size for this current study was determined utilizing G* Power 3.1.9.4 (Heinrich-Heine-Universität, Düsseldorf, Germany). In the ANOVA analysis, sample sizes of 10 premolars were derived for each of the 3 groups, resulting in a total sample of 30 subjects. This configuration yields an effect size (f) of 0.52, and 85% power to discern disparities at a significance level of 0.05.

### Sample selection and preparation

This study involved thirty recently extracted intact single-rooted mature permanent human mandibular first and second premolars that were extracted for orthodontic reasons. The teeth were examined under a magnification lens (8X) (Labomed*®* Magna dental microscope) that allowed observing the absence of cracks, imperfections, or apical resorptions. After extraction, any visible calculus was removed using ultrasonic tips (Eighteeth, Changzhou, China), then teeth were kept for 2 h in 4% NaOCl (Shahabamed, Aleppo, Syria), and then stored in normal saline [19]. Two periapical X-rays (buccolingual and mesiodistal) were taken to each premolar to ensure that it was free of anatomical abnormalities. Premolars with radicular resorption, root curvature, immature apex, fracture, or previous obturation were excluded.

The selected premolars were decoronated at 12 mm from the apex by a double-faced diamond disk (KG Sorensen, Cotia, Sao Paulo, Brazil), mounted on the low-speed straight handpiece to standardize sample lengths and facilitate canal instrumentation [20].

**Fig. 1:**
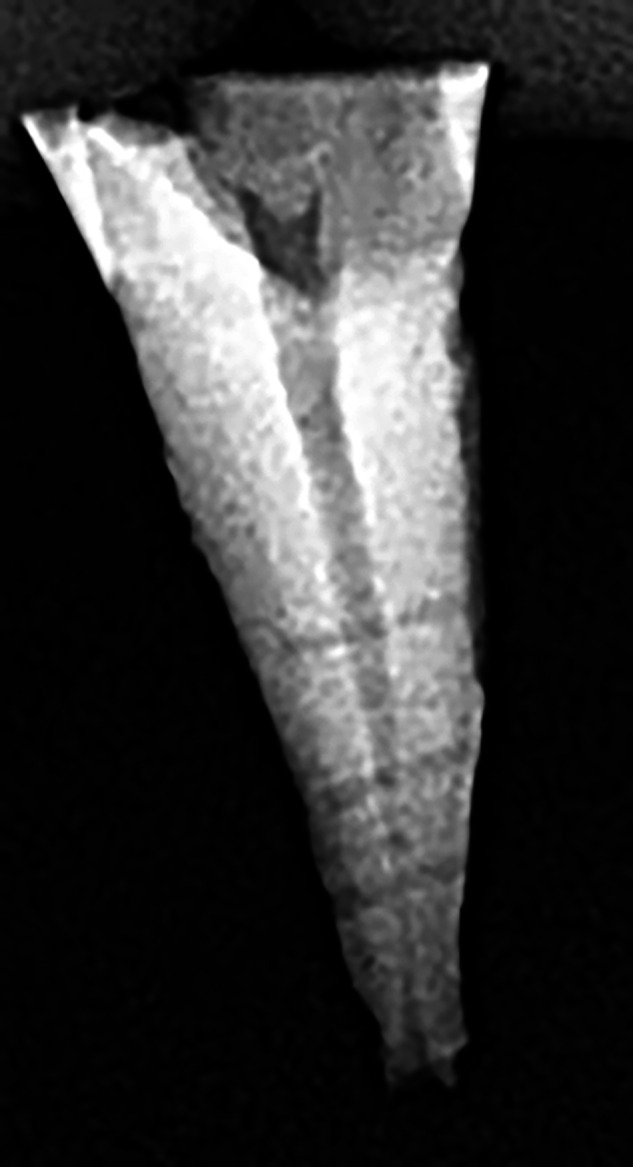
Simulated lateral canals as shown in the periapical radiograph. Lateral canals as shown in the periapical radiograph.

Afterward, they were prepared manually at a working length (WL) of 11 mm up to #15 K-file (Fanta Dental Materials, Shanghai, China) and then up to #30 taper 4% (30.04) using the F-one rotary system (Fanta Dental Materials, Shanghai, China). Each canal was irrigated with 15 ml of 5.25% NaOCl during instrumentation. Afterward, six lateral canals were created in pairs at 3, 5, and 7 mm from the root apex using #10 modified C-file (MC) (Fanta Dental Materials, Shanghai, China). This instrument was modified to be suitable for the low-speed handpiece, where the file handle was modified, and the instrument was fixed with cyanoacrylate to a mandrel [20, 21]. The MC was mounted at a low speed, and then under copious situation refrigeration, the tip of the MC was carefully inserted into the external root surface until the desired root channel was formed in both the mesial and distal sides of the root [22]. Thus, a total of 180 simulated lateral canals were obtained (6 canals/tooth, 2 lateral canals at each level) within uniform size (#10) (Fig. 1).

The final irrigation was done by a 27-gauge irrigation needle using 5.25% NaOCl for 20 min followed by 17% EDTA for 1 min. Finally, a final flush with 5.25% NaOCl was used for 1 min. After using each irrigant, canals were irrigated with normal saline for 1 min to eliminate the residual effect of the different irrigation solutions [23]. Finally, each root was covered with soft modeling wax (Cera Reus SA, Reus, Spain) and embedded in an acrylic block (5 × 5 × 15 mm) to simulate the periodontium. During this procedure, a standard 30.04 master gutta-percha cone (DiaDent, Cheongju, Korea) was introduced into the canal to the working length to prevent wax penetration into the canal space, then it was removed [24]. In the next step, CBCT images were taken using a papaya 3D plus (Genoray, Korea) at a setting of 85 Kvp voltage, 9 mA milliamperage, 14S exposure time, 10 × 15 cm field of view, and 0.1 mm voxel size. The teeth were meticulously arranged in sequential order on a custom-made plastic holder for precise identification and localization, allowing for CBCT imaging before obturation.

### Root canal obturation

Each canal was dried using (30.04) paper points and was individually filled. Subsequently, samples were randomly assigned to three groups based on the obturation technique used: group 1 was filled using the Cold Lateral Compaction (CLC) technique, group 2 was filled using the Continuous Wave Compaction (CWC) technique, and group 3 was filled using the Single Cone (SC) technique (*n* = 10 for each group). For all groups, a standard 30.04 master gutta-percha cone (DiaDent, Cheongju, Korea) was selected for each premolar, ensuring it demonstrated tug-back at the working length. Additionally, BCHiF Sealer (Brasseler USA, Savannah, GA, USA) was injected into the root canal up to 4 mm short of the WL using plastic syringes and needles provided by the manufacturer for all groups.

### Cold lateral condensation technique groups

A spreader (25.02) was pre-fitted to ensure that it could be inserted into 1–2 mm before the working length. After injecting the BCHiF Sealer, a standard CLC technique was carried out, where the spreader was placed into its maximum depth, and removed by rotating it back and forth as it was withdrawn [25]. The accessory cones (20.02, and 15.02) were placed in the space vacated by the spreader. The process was repeated until the spreader no longer went beyond the coronal third of the canal.

### Continuous wave compaction technique groups

The Fast-Pack tip was pre-fitted to reach 3 mm before working length and the device temperature (Fast-Pack; Eighteeth, Changzhou, China) was set at 200 ^o^C. After injecting BCHiF Sealer, 0.5 mm of the master cone was clipped to avoid exceeding the thermoplastic gutta-percha outside the apex, then it was inserted into the canal, and then the Fast-Pack tip was inserted with a one-way motion, and taken to a depth of 3-mm before the WL. The tip was allowed to cool for 15 s, then a single burst of heat was applied for 1 s, and then the tip was removed. The canal was backfilled with a Fast-Fill device (Fast-Fill; Eighteeth, Changzhou, China), where it was set at 180 °C and expressed in one continuous movement until the complete obturation of the canal with gutta-percha. Then, the excess gutta-percha was removed immediately from the canal orifice and compacted with a hand plugger (Dentsply, Tulsa, OK, USA) [26].

### Single cone technique

After injecting BC Sealer HiFlow, the master cone was slowly inserted into the canal to its working length. Then, the excess GP was trimmed off with an electrical heat carrier 1 mm below the orifice and vertically compacted with a hand plugger [27].

### CBCT evaluation

**Fig. 2:**
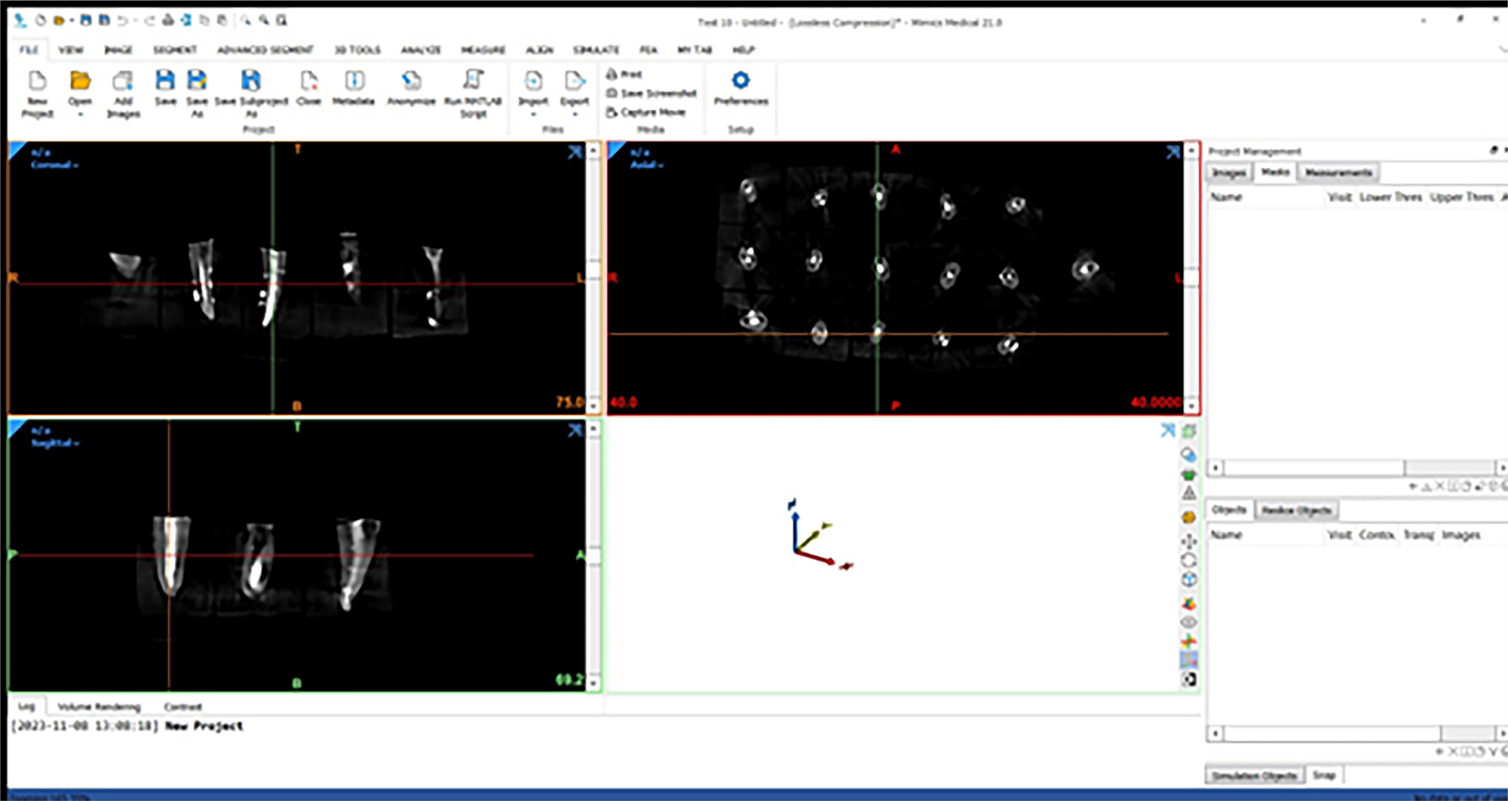
Openning The DICOM file of the studied sample using the Mimics Research program. The DICOM file on the Mimics Research program.

After completing the obturation procedures, the teeth were carefully restored to their original positions on the holder for post-obturation CBCT scans with the same alignment, arrangement, and radiation settings as the sample before obturation. This was done to enhance the credibility of comparing images before and after the obturation process. Subsequently, the CBCT Digital Imaging and Communications in Medicine (DICOM) files were opened in 3D medical imaging processing software (Mimics Research v21.0.0.406; Materialise NV, Leuven, Belgium), where the two lateral canals volume at each level were analyzed before and after obturation using this software in the same mechanism (Fig. 2).

**Fig. 3:**
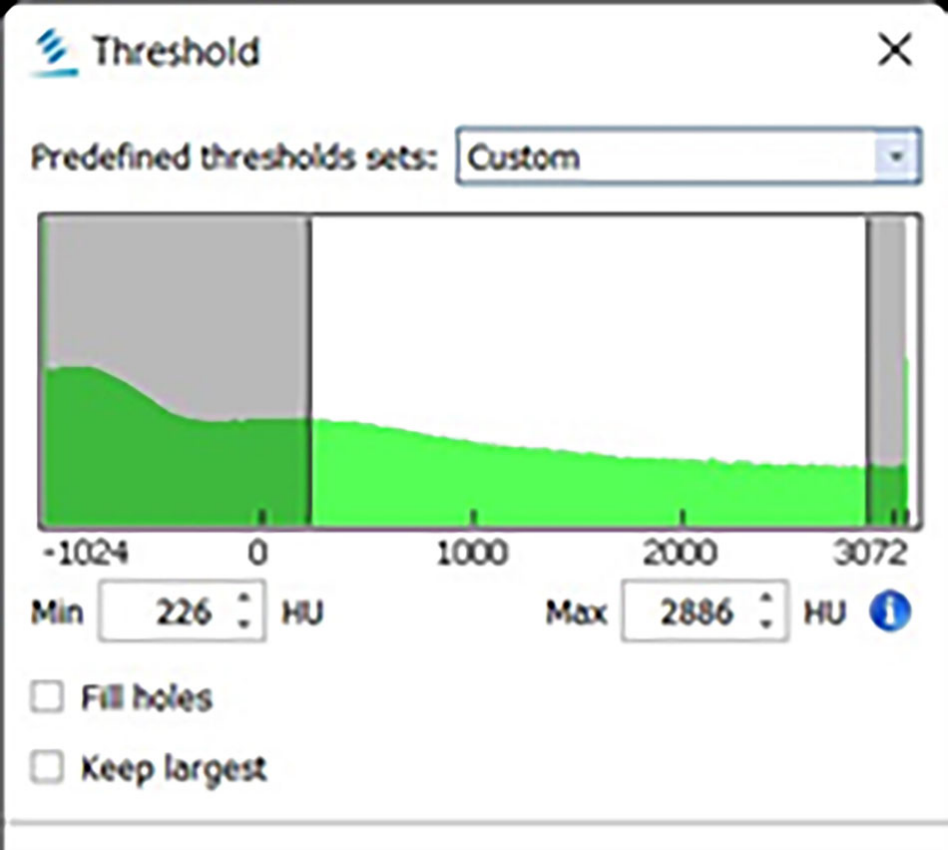
Hounsfield scale window in the Mimics Research program that used to determine the density of the air/sealer material in the lateral canals. Hounsfield scale to determine the density of the air/sealer material in the lateral canals.

The lateral canal volume was determined at each level using the semiautomatic segmentation process. A mask, which is an adjustable highlighted area on the image slices, was used as a tool to segment out lateral canals at each level slice by slice. Initially, a mask was created by estimating the Hounsfield (Fig. 3).

**Fig. 4:**
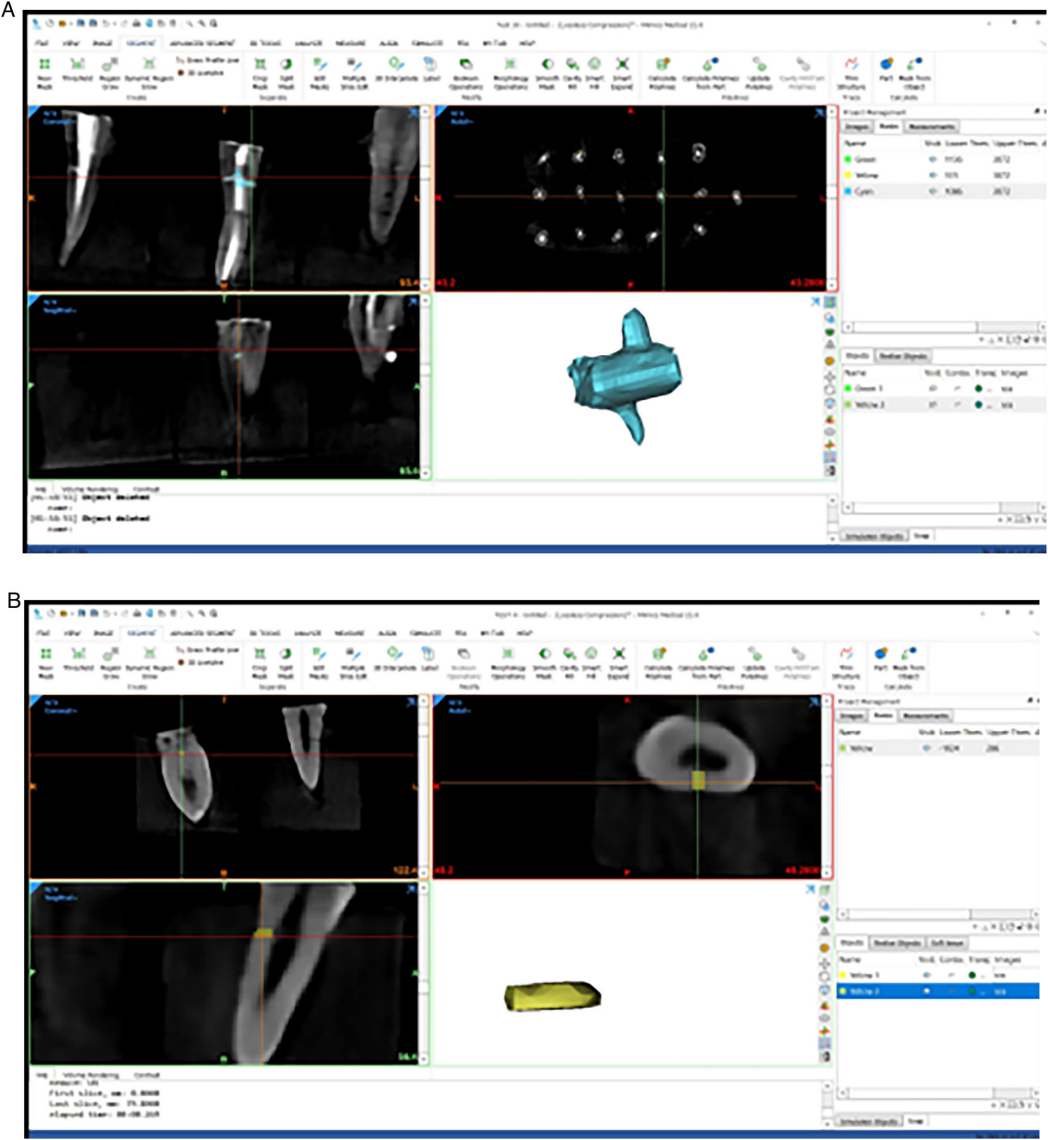
Isolation the lateral canal space in the STL format using the Mimics Research program. Isolation the lateral canal in the STL format **A** Determining the maximum diameter of the lateral canal. **B** The coronal view, the axial view, the sagittal view, and the 3D representation of the lateral canals in the STL format.

The mask was subsequently cropped in the coronal, sagittal, and/or axial planes to eliminate extraneous areas. Manual refinements were applied to the mask to include relevant areas in the segment and exclude irrelevant ones. These adjustments were meticulously performed slice by slice across the images in all three planes to ensure precise delineation of the lateral canal borders and accurate segmentation of each lateral canal slice (Fig. 4A). The segmented mask of the lateral canal was then removed from the remainder of the CBCT data and converted into a stereolithography (STL) file (Fig. 4B).

This STL file allowed for the visualization of lateral canals in 3 dimensions and was used to check for the presence of any voids or irregularities. The volume, determined by the Materialise Mimics software, was then recorded using the automated features in the software.

Finally, the 3D lateral canal obturation volume was compared to its unfilled volume to get the obturation percentage according to the following formula {(the volume of unfilled canal − the volume of filled canal) × 100/the volume of unfilled canal}.

The previous analysis method, using CBCT and MIMICS software, was similar to the one described by Sharki and Ali [18].

### Statical analysis

The collected data were tabulated and analyzed using SPSS software (Version 20, IBM SPSS Inc., Chicago, IL, USA). Kolmogorov–Smirnov test and Shapiro–Wilk test indicated normal distribution of the 3D lateral canals obturation volume percentage among the three groups at each level (*P* > 0.05). Moreover, homogeneity of variance was assessed by using Levene test which indicated that variances were homogeneous among groups (*p* > 0.05), so the comparison between groups regarding 3D lateral canals obturation volume percentage was performed using the One-way ANOVA test, and the pairwise comparisons were performed using the Sidak test. The level of significance was set at α < 0.05.

## Results

The quantitative outcomes detailing the volume percentage of 3D lateral canal obturation from the initial CBCT image analysis, including the volume of lateral canals before treatment in mm^3^, along with the results of the One-way ANOVA test, are depicted in Table 1.

**Table 1:** Mean and standard deviation of lateral canals volume before obturation at each level (3 mm, 5 mm, and 7 mm from the apex) among groups.

Canal volume before obturation				
Level/Group	CLC group	CWC group	SC group	*P* value^a^
	Mean ± SD	Mean ± SD	Mean ± SD	
At 3 mm level	0.25 ± 0.010	0.25 ± 0.015	0.25 ± 0.017	0.999
At 5 mm level	0.51 ± 0.007	0.50 ± 0.010	0.50 ± 0.011	0.999
At 7 mm level	0.84 ± 0.020	0.85 ± 0.010	0.84 ± 0.013	0.999

*SD* standard deviation.

^a^One-way ANOVA test

The One-way ANOVA test showed no significant differences between lateral canals volume at each level before obturation among the groups, indicating that the lateral canals volume before the obturation was uniform among the study groups.

The quantitative results of the 3D lateral canals obturation volume percentage of the second CBCT image analysis in terms of 3D lateral canals obturation volume percentage and the One-way ANOVA test result are presented in Table 2.

**Table 2:** Mean and standard deviation of 3D lateral canals obturation volume percentage at each level (3 mm, 5 mm, and 7 mm from the apex) among groups.

3D lateral canals obturation volume percentage				
Level/Group	CLC group	CWC group	SC group	*P* value^a^
	Mean ± SD	Mean ± SD	Mean ± SD	
At 3 mm level	69.88% ± 23.4	95.06% ± 2.6	37.87% ± 24.6	<0.001^b^
At 5 mm level	67.86% ± 25.8	67.14% ± 24.5	26.87% ± 20.4	0.003^b^
At 7 mm level	42.50% ± 21.3	56.97% ± 22.8	18.80% ± 17.1	0.005^b^

SD: standard deviation.

^a^One-way ANOVA test.

^b^Significant differences.

The One-way ANOVA test showed significant differences between 3D lateral canals obturation volume percentage at 5 mm and 3 mm levels among the groups (*P* = 0.003 and *P* < 0.001; respectively).

The Sidak test was used to detect differences in pairwise comparisons, which showed that, at the 3 mm level, the CWC group had the highest mean of 3D lateral canals obturation volume percentage ($\bar{x}$ = 95.06%) compared with that of the CLC and SC groups, and the differences were statistically significant ($\bar{x}$  =  69.88% and $\bar{x}$  =  37.87%; *P*  =  0.054 and *P*  < 0.001, respectively). Moreover, the CLC group had a higher mean of 3D lateral canals obturation volume percentage (*P* = 0.012). Additionally, at the 5 mm level, the SC group had the lowest mean of 3D lateral canals obturation volume percentage ($\bar{x}$ = 26.87%) compared with that of the CWC and CLC groups, and the differences were statistically significant ($\bar{x}$  =  67.14% and $\bar{x}$  =  67.86%; *P*  =  0.008 and *P*  =  0.007, respectively).

Additionally, at the 7 mm level, the SC group had the lowest mean of 3D lateral canals obturation volume percentage ($\bar{x}$ = 18.80%) compared with that of the CWC and CLC groups, and the differences were statistically significant ($\bar{x}$  =  56.97% and $\bar{x}$  =  42.50%; *P* =  0.004 and *P* =  0.042, respectively).

## Discussion

In endodontic practice, the complex anatomy of root canals significantly impacts the disinfection and obturation of lateral canals, particularly in the middle and apical thirds of the root [1–6]. Effective treatment of lateral canals is crucial to prevent bacterial growth and reinfection of the root canal system [28]. The type of sealer, its physical and chemical properties, and the obturation technique are critical factors affecting lateral canal obturation [29–32]. Thus, this study aimed to identify the optimal obturation techniques using BCHiF sealer to effectively seal lateral canals at various levels.

While the obturation of natural lateral canals in extracted teeth has been examined [30], the challenges in obtaining human teeth with natural branches led to the creation of artificial lateral canals [31, 32]. These simulated lateral canals closely mimic clinical and anatomical conditions, with diameters comparable to those reported in previous studies [2, 33].

Human teeth were preferred over clear acrylic for experimental simulation because the acrylic formulation differs from natural teeth in terms of surface structure, with acrylic hardness ranging between 20-22 kg/mm² and dentin hardness between 35-40 kg/mm² [34]. Additionally, resin blocks present certain limitations, such as the absence of a smear layer, variations in surface texture, and resin condition, which may either enhance or impede the flow properties of the sealer [35].

The simulated lateral canals were prepared after shaping the main canal to minimize the risk of altering the axis of the lateral canals and to avoid penetration into the main canal. A layer of grade wax was applied to the external root walls to simulate the periodontal ligament found in natural teeth. This layer is crucial because the absence of it can affect the insertion of the filling material into the lateral canals due to differences in pressure within the canal and the ease with which the material can exit the lateral canal during obturation.

Sodium hypochlorite was used as an irrigant due to its availability and common use in endodontics. A 5.25% concentration was chosen because it is widely used in endodontic treatments and is effective at dissolving both vital and necrotic pulp tissue and the biofilm layer within the root canal system [36]. The smear layer formed during canal preparation can inhibit the penetration of irrigants, medicaments, and sealers into dentinal tubules, which can interfere with sealer penetration during obturation. Therefore, removal of the smear layer before obturation is highly recommended [37]. A 17% EDTA solution was employed as a chelating agent to remove the smear layer due to its ability to dissolve the inorganic components by removing calcium ions from the dentin [38]. Eskander et al. demonstrated that the chelating action of EDTA significantly enhances the penetration of Bioceramic sealer into dentinal tubules [39].

The cold lateral condensation (CLC) technique was evaluated as it is a classic obturation method primarily taught in undergraduate dental programs [40]. The single cone (SC) technique was also assessed, as the manufacturer suggested that the BCHiF sealer could be used with the SC technique to achieve results comparable to other obturation methods. The SC technique is gaining popularity due to its increasing use with rotary file preparation, its simplicity, and its ability to enhance clinical efficiency while reducing the effort for both the practitioner and the patient [41].

CWC technique was also assessed, which relies on softening the obturation material. Notably, obturation techniques that thermally soften gutta-percha tend to yield better results for filling anomalies and secondary canals compared to cold obturation techniques [31]. The current findings are consistent with these previous observations.

The quality of the endodontic sealer is crucial for the effective obturation of lateral canals and ramifications. Therefore, the BCHiF sealer was selected for this study due to its high flow properties and compatibility with thermal obturation techniques [42].

To evaluate the extent of lateral canal obturation in natural teeth, radiographic evaluation, clearing techniques, or both have been employed [31, 32]. However, Clark and Eldeeb found that lateral and accessory canals obturated on cleared teeth did not always appear in radiographic images [43]. Similarly, Almeida et al. reported that radiographs failed to show lateral canal obturation in about 8% of cases [44]. Moreover, Matherne et al. demonstrated the superiority of CBCT over conventional radiography in detecting supplemental canals [45]. Consequently, CBCT images were taken before and after the obturation stage in this study to maximize the diagnostic benefit and establish a new method for evaluating the quality and percentage of root canal system obturation using volumetric analysis [46].

In this study, the use of DICOM file conversion to create three-dimensional models, along with the volumetric analysis capabilities of MIMICS software, provided significant advantages. This approach has been successfully applied in similar studies to generate detailed three-dimensional models [18, 47].

It is noteworthy that the volumes of the empty simulated lateral canals differed at the 5- and 7-mm levels compared to the 3-mm level. This discrepancy is attributed to the increasing length of the lateral canals as they extend towards the coronal part of the root, despite using the same size MC files to create these lateral canals.

In this study, the null hypothesis was rejected, as it was observed that the 3D lateral canals obturation volume percentage is affected by the obturation technique and the location of lateral canals themselves. The CWC and CLC techniques showed the best volume percentage compared with the SC technique at the three levels. These findings met the observation of Buchanan [48] and Wolf [49]. These results may be attributed to the fact that the higher the force applied, the lower the viscosity and the greater the flow of the sealer. Although the force application is not continuous, it is a punctual force that is dissipated by the decrease of the pressure on the walls by increasing the taper, as well as by the loss of mass of the cement, resulting in the reduction of the flow and incomplete obturation of the lateral canals [49, 50].

On the contrary, the SC technique had the lowest 3D lateral canals obturation volume percentage, which met with Fernández and colleagues [5], and Yang and colleagues [12], but it differed from the study of Iglecias and colleagues [51], the difference may be attributed to use a different sealer within curved canals, as the penetration depth and fluidity of the sealer were not studied.

CWC technique is a simple and acceptable method providing better adaptation of gutta-percha and sealer to the entire canal walls especially the apical area [52], On the contrary in the CLC technique, the voids were seen between accessory cones because they did not fully occupy the space created by the spreader [53], this explains the superiority of the CWC technique in sealing the lateral canals at 3 mm level.

Robberecht et al. reported that the quality of the obturation with warm gutta-percha was better than that of the tapered single-cone technique regarding apical leakage, gutta-percha adjustment, and filling of lateral/accessory canals. In this last aspect, their findings were similar to those reported in this study as both groups, the CWC and CLC techniques, had higher acceptable obturation rates [54].

In the SC technique, a cold gutta-percha point is inserted into the canal and the sealer fills the irregularities. Somma and colleagues [55] and Wu and colleagues [56] found that this technique requires more sealer volume than other obturation techniques.

Marciano et al. reported that at 2 mm from the WL, the gutta-percha and sealer percentages were similar between the CWC and SC techniques. This may be attributed to the use of a different sealer (epoxy resin), and this affects the amount of sealer flow and its dependence on the activation of irrigants [57].

Moreover, this study diverged from the findings of Wolf and colleagues, who asserted the superiority of the CWC technique over the CLC technique, albeit not at the apical level [49]. This discrepancy may be attributed to the use of acrylic specimens instead of natural teeth, as well as variations in the type of sealer employed.

The main limitation of this study lies in the fact that the root canal system, which includes lateral canals, may be more complex than the relatively simple model adopted in the current study. Further studies with a similar methodology should be conducted on teeth with more complex anatomical shapes and possibly real lateral canals.

Further studies that include combining several obturation techniques and irrigation activation systems are required to assess the best protocol to treat lateral canals. There is also a need to compare the results of this newly presented evaluation method with other methods, especially with the confocal laser scanning microscopy, and assess the previously presented method with micro-CT images instead of CBCT images to reach more accurate results and evaluation. Furthermore, additional studies are needed to correlate the 3D assessment of root canal obturation with apical sealing provided by root canal filling materials.

## Conclusions

Within the constraints of this investigation, the application of BCHiF alongside the CWC and CLC techniques demonstrated elevated volume percentages of obturation within lateral canals in comparison to SC technique. This suggests potential enhancements in lateral canal obturation quality and consequently, the overall efficacy of endodontic therapy for teeth featuring lateral canals. The proposed MIMICS software will allow clinicians to accurately classify sealing rates according to their volume, allowing for better prediction of treatment outcomes in teeth.

## Data Availability

De-identified data are available upon reasonable request to the corresponding author.
